# Single Nucleotide Polymorphisms and Their Association with Coronary Artery Aneurysms and IVIG Resistance in Kawasaki Disease in Ireland

**DOI:** 10.1007/s00246-025-03989-0

**Published:** 2025-08-08

**Authors:** S. M. Duignan, K. Brennan, E. Connolly, A. Watson, D. Noone, E. Dunne, P. Gavin, A. Flinn, C. Ó′Maoldomhnaigh, S. L. Doyle, C. J. McMahon

**Affiliations:** 1 Department of Paediatric Cardiology, Children’s Health Ireland at Crumlin, Dublin, Dublin 12 Ireland; 2 Department of Clinical Medicine, School of Medicine, Trinity College Dublin, Dublin, Ireland; 3 Department of Paediatric Infectious Diseases, Children’s Health Ireland at Crumlin, Dublin, Ireland

**Keywords:** Kawasaki disease, SNP, Coronary artery aneurysm, IVIG resistance, Genetics

## Abstract

Kawasaki disease (KD) is the leading cause of acquired heart disease in children in developed countries. The exact aetiology remains a mystery but genetics play an important role. Treatment with intravenous immunoglobulin (IVIG) successfully reduces the prevalence of coronary artery aneurysm (CAA) formation, however, IVIG resistance affects 10–20% of patients. We aimed to assess whether single nucleotide polymorphisms (SNPs) which have been associated with KD in other populations are also relevant in our national cohort. Samples were collected via buccal swabs and genotyping was performed using KASP Genotyping Assay at LGC Genomics. Chi-square (*χ*^2^) analysis or *χ*^2^ analysis for trend were used to examine genotype distributions and allele frequencies. Numerical variables were compared between two groups using the Mann–Whitney *U*-test and the Kruskal–Wallis test for groups of 3 or more. Of the 39 patients included, the majority were cases of typical KD (82%). A third developed CAA and 31% demonstrated IVIG resistance. Participants harboring the AA genotype of *VEGFA* rs699947 had a significantly reduced association with coronary lesions and IVIG resistance. Analysis of the *CD40* rs1569723 (A/C) revealed an increased risk associated with the C allele for coronary artery lesions and IVIG resistance. We performed the first evaluation of the genetic profile of patients with KD in Ireland. We found that the *VEGFA* rs699947 C/A polymorphism is protective while the *CD40* rs1569723 A/C polymorphism harbors increased risk. This study is the first to correlate the *VEGFA* rs699947 polymorphism with KD outcomes in the literature.

**Supplementary Information:**

The online version contains supplementary material available at 10.1007/s00246-025-03989-0.

## Introduction

Kawasaki disease (KD) is an acute inflammatory syndrome of childhood associated with a predominantly medium-vessel vasculitis with a predilection for the coronary arteries [1, 2]. It is usually self-limiting, however coronary sequelae can lead to early and late complications including myocardial infarction and death, and in developed countries, KD is now the leading cause of acquired heart disease in children [3].

Despite intense study for decades, the aetiology of KD remains a mystery. A broadly accepted hypothesis is that KD occurs due to an abnormal immunologic reaction in genetically predisposed children exposed to an infectious or environmental trigger [4, 5]. The importance of genetics is demonstrated by the fact that family history in a first degree relative is a risk factor for KD [6, 7], and the higher incidence of KD amongst certain ethnicities which persists even after geographic relocation (exemplified by those of Japanese descent living in Hawaii [8]).

Coronary arteritis leading to coronary artery aneurysms (CAA) occurs in a quarter of untreated patients with KD [1]. In patients with large CAA following an episode of KD, the ten-year cardiac event-free survival rate is approximately 50% in male patients and 75% in female patients [9]. Treatment with intravenous immunoglobulin (IVIG) successfully reduces the prevalence of CAA formation to 5% [10]. However, IVIG resistance, variably defined in the literature as recrudescent or persistent fever 24–48 h following first IVIG infusion, affects 10–20% of patients [1, 11–15], in whom the risk of CAA development is significantly increased [1, 16, 17]. Early treatment intensification in those at high risk of IVIG resistance may improve outcomes [18]. Although multiple prediction scores have been created, a recent meta-analysis concluded that none of these accurately distinguish between patients with and without IVIG resistance [19]. A priority of ongoing research is therefore to identify patients at risk of IVIG resistance who may benefit from early treatment intensification with drugs such as corticosteroids, infliximab, anakinra, and/or ciclosporin.

The genetics of KD have never been studied in a population in Ireland. We therefore sought to assess whether single nucleotide polymorphisms (SNPs) which have been associated with Kawasaki disease in other populations are also relevant in our national cohort [20–32]. We chose SNPs which were associated with an increased susceptibility to KD, an increased risk of coronary artery aneurysms, and an increased risk of resistance to IVIG.

## Materials and Methods

### Study Population

The study population included patients with a diagnosis of typical or atypical Kawasaki disease who were seen at the national children’s hospital (Children’s Health Ireland at Crumlin, Dublin) in Ireland.

Patients were identified from the infectious disease departmental database, which included patients diagnosed between January 2010 and December 2014, and the echocardiography database which included patients since its inception in 2009 up until March 2023. We excluded those who presented during the COVID-19 epidemic to avoid contamination of the study population with patients who potentially had Paediatric Multisystem Inflammatory Syndrome temporally related to COVID-19 (PIMS) with a Kawasaki-like presentation as the relationship between KD and PIMS is incompletely understood, with ongoing debate in the literature regarding whether they represent two distinct clinical entities or a syndrome continuum [33, 34]. Inclusion criteria included a diagnosis of KD or atypical/incomplete KD presenting during the defined study period. Exclusion criteria included the presence of a known condition affecting the immune system or presentation during the COVID-19 epidemic in Ireland. A letter was sent to eligible patients with study information, buccal swabs and instructions and patients were recruited and consented with a follow-up phone-call. IVIG resistance was defined in this study as persistent or recurrent fever (≥ 38 °C) 36 h after completion of IVIG infusion.

### Coronary Artery Assessment and Classification

Coronary artery dimensions were based on 2D echocardiography measurements from inner-edge to inner-edge in the parasternal short axis view of the left main coronary artery, left anterior descending artery, left circumflex artery and right coronary artery.

In more recent years in our centre, the AHA classification according to body surface area (BSA)-adjusted *z* score has been used [1]. According to the AHA classification, coronary artery dilatation is defined as a z score of ≥ 2 to < 2.5; a small aneurysm as a z score of *z* ≥ 2.5 to < 5; a medium aneurysm as a *z* score of *z* ≥ 5 to < 10 and absolute diameter < 8 mm and a large/giant aneurysm as a *z* score of ≥ 10 or absolute diameter ≥ 8 mm. However, in the earlier era, before widespread adoption of the *z* score classification in our centre, the Japanese Ministry of Health Criteria were used [35]. These criteria classify coronary arteries according to absolute dimension as abnormal if: > 3 mm in children < 5 years old, > 4 mm in children ≥ 5 years old, or if the internal diameter of a segment measures ≥ 1.5 times that of an adjacent segment. The Japanese Circulation Society classification was used to determine the size of the aneurysm as small (diameter ≤ 4 mm, or in children ≥ 5 years of age, lesions with an internal diameter < 1.5 × that of an adjacent segment); medium (> 4 mm and < 8 mm, or in children ≥ 5 years of age, lesions with an internal diameter 1.5–4 × that of an adjacent segment) or large (≥ 8 mm, or in children ≥ 5 years of age, lesions with an internal diameter > 4 × that of an adjacent segment) [36]. We defined coronary dilatation or aneurysm as persistent if it was still present on a follow-up scan at 4–6 weeks following initial treatment.

### DNA Isolation and Genotyping

Samples were collected via buccal swabs (Isohelix, SK-2S) and stored in BuccalFix DNA Stabilisation Buffer (Isohelix, BFX-25) at – 80 °C until DNA isolation following manufacturer’s specifications. Genotyping was performed using KASP Genotyping Assay at LGC Genomics for quality assurance, two ‘no template’ controls were included. 30 SNPs were initially examined with 23 validated for analysis. Seven SNPs were removed from the analysis due to zero counts for genotypes. KASP genotyping was not validated by Sanger sequencing, as KASP is designed for known SNPs and has very low error rates. The KASP assays were internally quality controlled, and the results reported showed clear genotype clustering and high call rates. Supplementary Table 1 includes the full list of SNPs and 50 bp flanking regions for KASP assay design. Hardy-Weinburg Equilibrium (HWE) was examined for all SNPs included in the analysis, with *CD40* rs1569723 falling out of HWE (*P* = 0.018). To assess whether SNPs in our population were relevant for susceptibility to KD, we compared the prevalence of these SNPs in our cohort compared to the to the Allele Frequency Aggregator (ALFA) dataset which contains allele frequency data from over 200,000 patients from diverse populations [37]. We compared our data to the European population dataset within ALFA. Note that patients who had not received IVIG were not included in the analysis of SNPs associated with IVIG resistance.

### Statistical Analysis

Data were analysed using SPSS Statistics 28 (IBM Corporation, Chicago, IL, USA) and GraphPad Prism software version 10 for Windows (GraphPad Software, San Diego, CA, USA). The data were presented as a proportion for categorical variables and mean ± standard deviation for continuous variables. Chi-square (*χ*^2^) analysis or *χ*^2^ analysis for trend were used to examine both genotype distributions and allele frequencies. Numerical variables were compared between two groups using the Mann–Whitney *U*-test and the Kruskal–Wallis test for groups of 3 or more. The magnitude of effect size was expressed as odds ratio with a 95% confidence interval (CI). A *P*-value of < 0.05 was considered significant.

### Ethics

This study received full approval from the ethics committee in Children’s Health Ireland at Crumlin (GEN/843/20).

## Results

### Patient Characteristics

There were 154 patients identified who fulfilled the inclusion criteria. Fifty-two patients consented to the study; however, 13 sample packs were returned which were unusable (10 = insufficient DNA, 2 = bacterial overgrowth, 1 = missing patient information). These patients were excluded resulting in 39 patients included in the final analysis (see Table 1). The majority of cases were male (*n* = 23, 59%) and classic presentations (*n* = 32, 82%), with just 7 cases of atypical Kawasaki disease. Patients ranged in age at presentation from 2 months to 10 years, with a median age of 37 months. Maximum CRP result was available for 35 patients, 4 patients who were initially managed at an outside referring hospital did not have CRP results available for the analysis. The median maximum CRP level was 160 mg/L (range 4 − 307 mg/L).

**Table 1:**
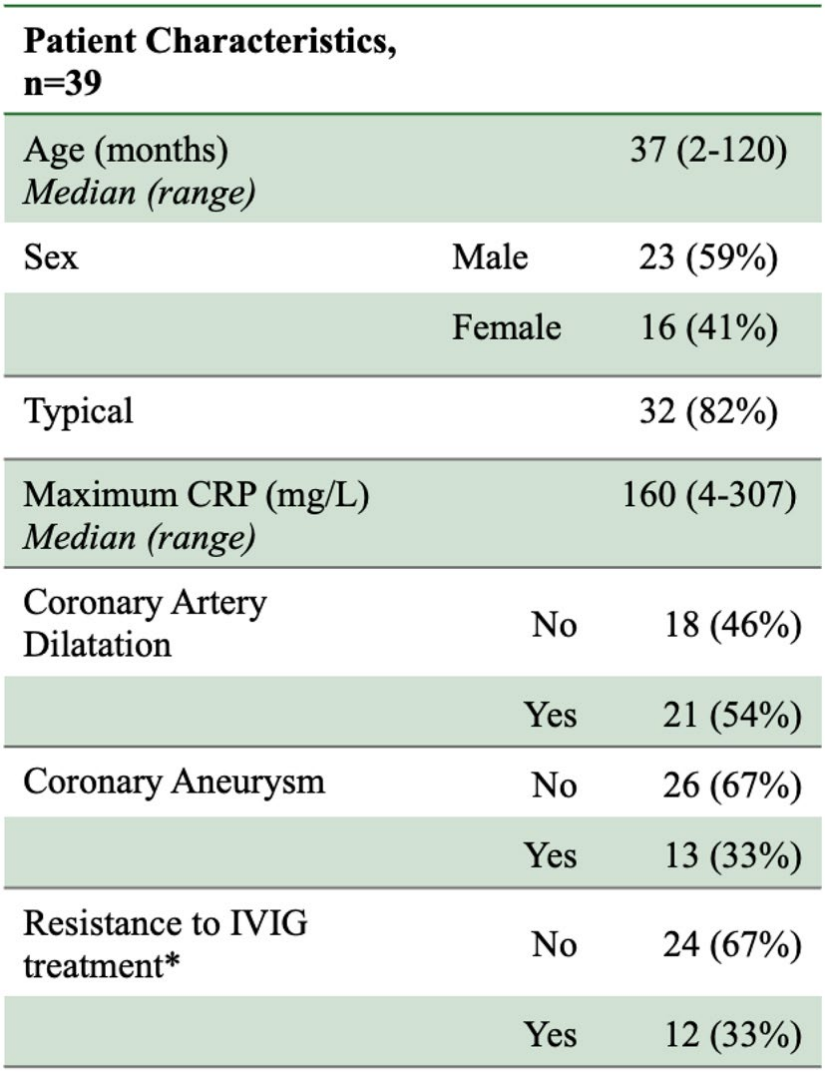
Patient characteristics

Data are expressed as frequency (percentage). IVIG, Intravenous Immunoglobulin

* 36 patients received treatment with IVIG

### Treatment

**Fig. 1:**
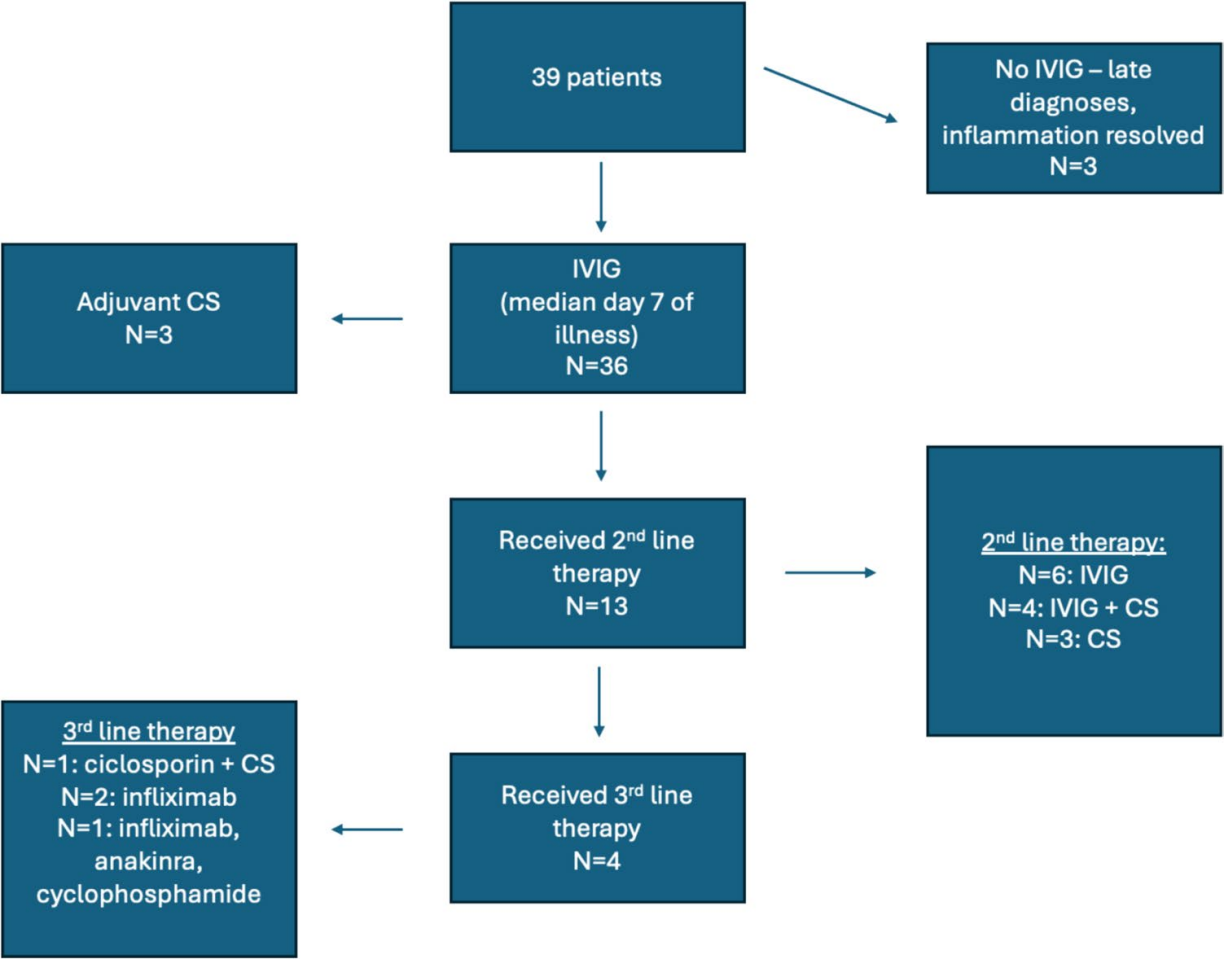
Treatment received

The majority of patients (36/39, 92%) received intravenous immunoglobulin 2 g/kg (IVIG) as first-line therapy, on a median of day 7 of symptoms (range day 3 – day 21, data unavailable for one patient treated initially in an external hospital). The three patients who did not receive IVIG were diagnosed late in the presentation of their illness, when fever and inflammation had resolved. Eleven patients received corticosteroids. In three cases, this was as adjuvant therapy in high-risk patients in conjunction with initial IVIG treatment, and in 8 cases this was part of second- or third-line therapy. The high-risk patients were under 6 months of age and had coronary artery aneurysms present at presentation. The protocol for corticosteroids was IV methylprednisolone 2 mg/kg once daily for 3–5 days followed by a switch to oral prednisolone (1-2 mg/kg/day, rounded to the nearest 5 mg dose) which was then weaned over 2–3 weeks. Of the 36 patients treated acutely, all received moderate to high-dose aspirin therapy (50-100 mg/kg/day) (median duration 1 week, range 1–6 weeks). Twelve of 36 (31%) patients demonstrated IVIG resistance with persistence or recurrence of fever and/or inflammation ≥ 36 h following the first dose of IVIG (median day 12 of illness, range day 5—day 28). Thirteen patients received 2nd line therapy as outlined in Fig. 1.

### Complications

Three patients (7.6%) had transient pericardial effusions at presentation which resolved spontaneously without medical intervention, and 8 patients (20.5%) had transient atrioventricular valvular regurgitation which resolved prior to discharge. Over half (*n* = 21, 54%) of patients had dilatation of one or both coronary arteries at presentation. In 7 cases, this resolved without progressing to aneurysm formation, whereas in 13 cases (33.3%), patients developed one or more coronary artery aneurysms (Table 2). One patient developed aneurysms in brachial and iliac vessels as well as giant coronary artery aneurysms. This patient’s course was complicated by a left main stem coronary artery thrombus which led to reduced left ventricular function.

**Table 2:**
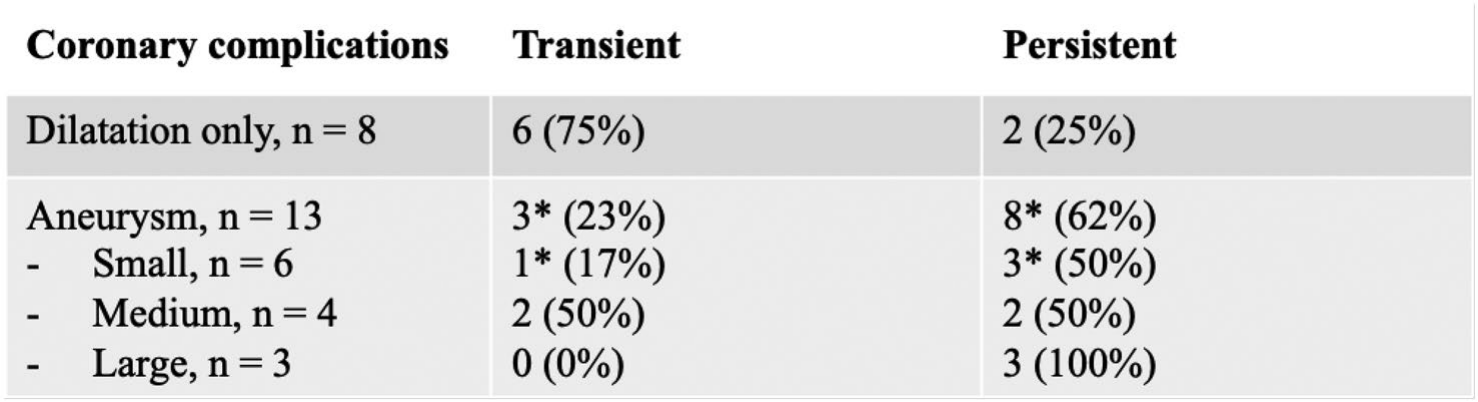
Coronary artery anomalies

*Data missing for two patients with small aneurysms

### Single Nucleotide Polymorphism Analysis

Of the 30 SNPs selected for investigation, 7 returned zero counts for genotypes and were excluded from further analysis. There was also a low call rate for some SNPs, likely due to low concentration or low quality of DNA in some patient samples, which precluded them from full analysis. For full SNP data, see Supplementary Table 1.

Regarding susceptibility, we compared the prevalence of SNPs in our cohort to that of the European dataset within ALFA. Four of the SNPs were significantly over-represented in our population, implying that they are associated with susceptibility in our patients (see Table 2): *ITPKC* rs2290692 (*P* value 0.0001), *SMAD5* rs10056474 (*P* value 0.0008), *CD40* rs1569723 (*P* value 0.0289), *CASP3* rs113420705 (*P* value 0.0314). Regarding clinical outcomes, of the 23 SNPs analysed, SNPs in *VEGFA* and *CD40* were associated with coronary artery lesions and IVIG resistance.

### VEGFA rs699947

Table 3 outlines the distribution of the *VEGFA* rs699947 (C/A) genotypes and allele frequencies by disease outcomes. Thirty-three of the 39 samples amplified for the *VEGFA* rs699947 genotype. Chi-square analysis found significant differences in genotype distribution associated with coronary artery dilatation (*P* = *0.013*) and IVIG resistance (*P* = *0.025*). Participants harboring the AA genotype of the *VEGFA* rs699947 had a significantly reduced association with coronary artery dilatation compared to CC carriers (OR: 0.11, *P* = 0.015) and CA carriers (OR: 0.15, *P* = 0.040). Homozygous carriers of the AA genotype also had a significantly reduced association with IVIG resistance compared to the CC genotype (OR: 0.1, *P* = 0.026). This was reflected in the analysis of allele distribution where presence of the rs699947 A allele was significantly associated with a reduced odds ratio for coronary artery dilatation (OR: 0.21, *P* = 0.002) and IVIG resistance (OR: 0.23, *P* = 0.009).

**Table 3:**
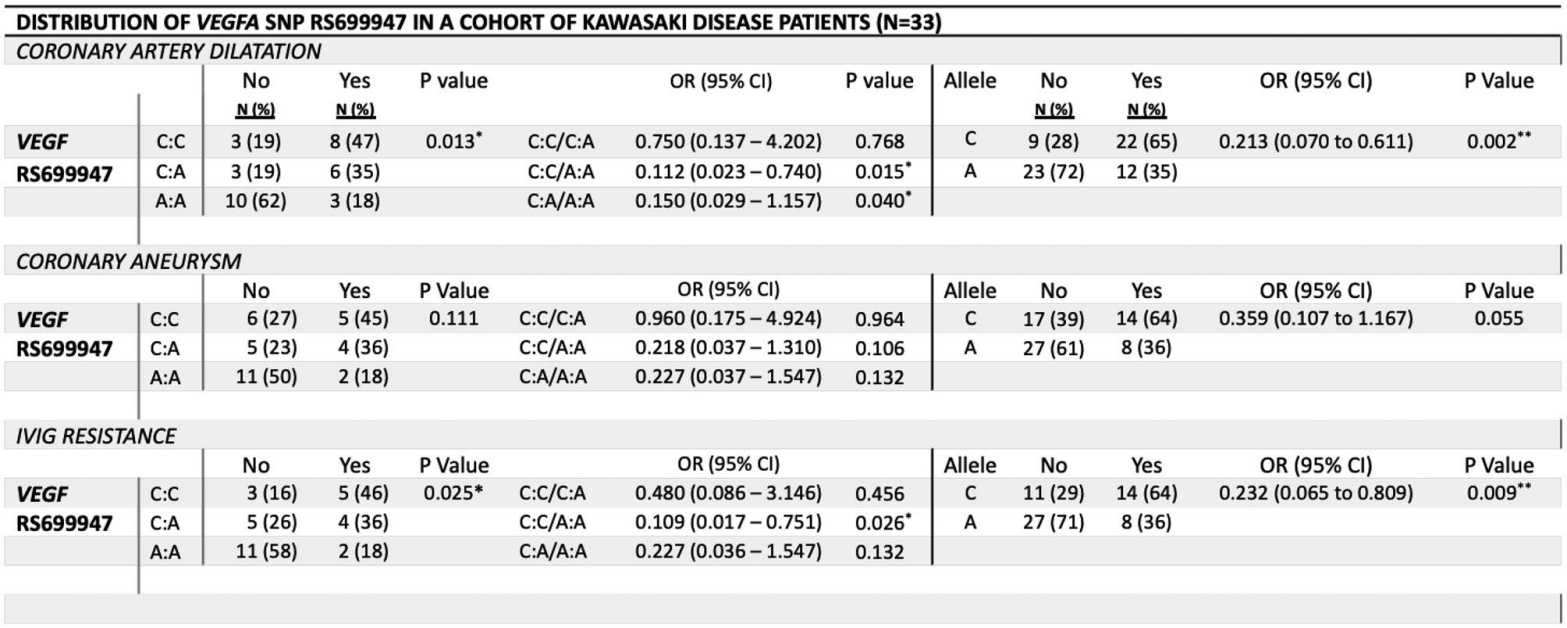
Distribution of *VEGFA* rs699947 in a cohort of Kawasaki disease patient in Ireland

Data are expressed as frequency (percentage). * denotes statistically significant P-values (*P* < 0.05). VEGF, Vascular endothelial growth factor, SNP, single nucleotide polymorphism; IVIG, intravenous immunoglobulin; OR, Odds ratio

### CD40 rs1569723

Analysis of the *CD40* rs1569723 (A/C) SNP, outlined in Table 4 found significant differences in the distribution of genotypes associated with coronary artery dilatation (*P* = 0.033) but not IVIG resistance (*P* = 0.054). There was an increased risk associated with the C allele for coronary artery dilatation (OR: 7.7, *P* = 0.006), coronary artery aneurysm (OR 4.2, *P* = 0.047) and IVIG resistance (OR: 7.8, *P* = 0.011). Upon genotype analysis, we found this effect was most significant between homozygous carriers of the A allele compared to homozygous carriers of the C allele for coronary artery dilatation (OR: 15.0, *P* = 0.030). Seventeen of the 39 samples amplified for the *CD40* rs1569723 genotype.

**Table 4:**
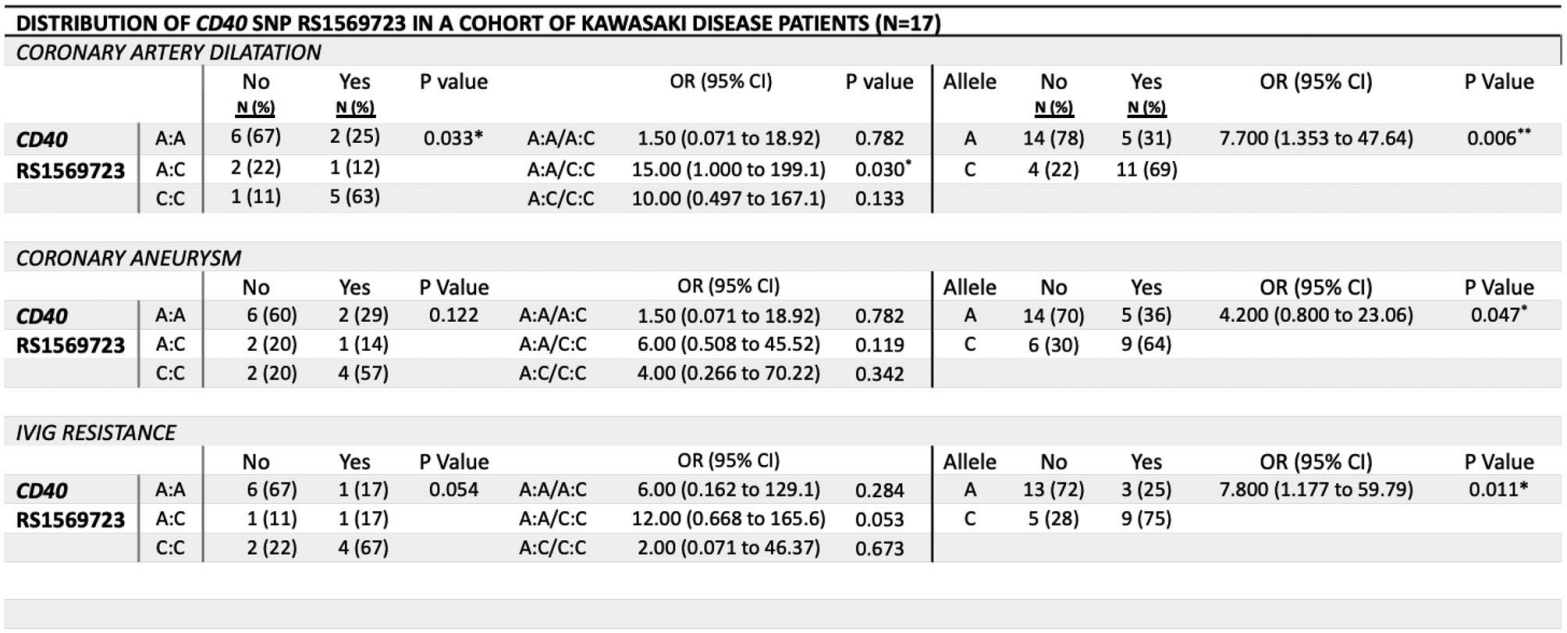
Distribution of *CD40* rs1569723 in a cohort of Kawasaki disease patients in Ireland

Data are expressed as frequency (percentage). * denotes statistically significant P-values (*P* < 0.05). CD40, Cluster of Differentiation 40, SNP, single-nucleotide polymorphism; IVIG, intravenous immunoglobulin; OR, Odds ratio

### CRP

**Fig. 2:**
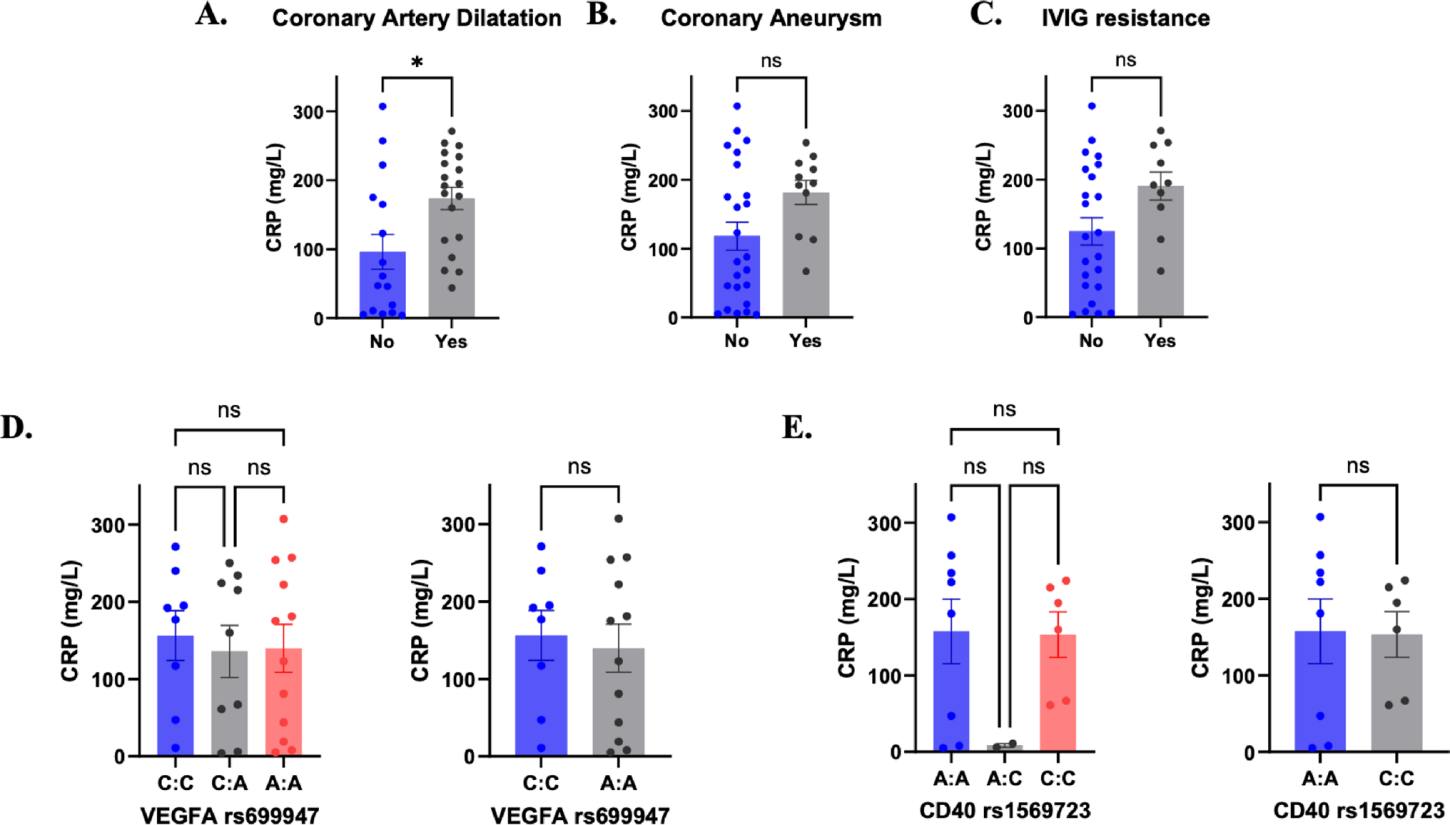
Relationship of maximum CRP level with coronary artery events, *VEGFA* rs699947 or *CD40* rs1569723 genotype. Circulating CRP levels were measured during initial presentation and diagnosis and correlation analysis performed investigating the relationship between maximum CRP level and coronary artery dilatation (**A**), coronary aneurysm (**B**) and IVIG resistance (**C**). Impact of *VEGFA* rs699947 allele homozygosity or heterozygosity on maximum CRP level (**D**). Relationship between *CD40* rs1569723 status on maximum CRP level at diagnosis (**E**). Bar graphs show mean ± SEM analyzed using the Mann–Whitney *U*-test or the Kruskal-Wallis test.

Significantly higher CRP levels were observed in patients with coronary artery dilatation (Fig. 2A), (173.4 ± 70.5 mg/L vs. 96.1 ± 100 mg/L; *P* = 0.011). Higher CRP levels were also noted in patients with coronary aneurysm (Fig. 2B) and IVIG resistance (Fig. 2C), however this did not reach statistical significance (*P* = 0.061 and *P* = 0.062, respectively).

We also examined the relationship between CRP levels and our SNPs of interest by *VEGFA* rs699947 (C/A) (Fig. 2D) and *CD40* rs1569723 (A/C) (Fig. 2E) genotypes and compared homozygous carriers of the reference and variants alleles. We did not observe significant differences in CRP levels between genotypes but did note that AA carriers of rs699947 had lower levels of CRP compared to CC carriers, though this was not statistically significant (AA: 139.7 ± 107.7 mg/L vs CC: 156.3 ± 91.00 mg/L; *P* = 0.678).

## Discussion

In this study, we investigated whether SNPs which have been associated with Kawasaki disease susceptibility, coronary artery aneurysm formation and IVIG resistance in other populations were relevant in a national cohort of patients with KD in Ireland. Four SNPs evaluated for susceptibility were significantly more prevalent in our KD patients compared to a large European reference cohort. There were significant findings relating to clinical outcomes in two of the polymorphisms examined. In the *VEGFA* rs699947 (C/A) SNP, carriers of an A allele harboured a decreased risk for both coronary artery dilatation and IVIG resistance. There was also a decreased risk of coronary artery aneurysms, though this failed to reach statistical significance (*P* = 0.055) which may have been related to the small sample size. Analysis of the *CD40* rs1569723 (A/C) SNP revealed an increased risk for coronary artery dilatation, aneurysms and IVIG resistance in those with a C allele.

### SNPs and Susceptibility

Four SNPs of those tested were significantly increased in prevalence in our cohort compared to a large European reference cohort, implying they are associated with susceptibility to KD in our population. In the case of *SMAD5* rs10056474, to the best of our knowledge, this is the first time this SNP has been associated with KD susceptibility in a European population. It is also interesting to note that 12 of the SNPs which we tested which had previously been associated with increased risk for KD in other populations, were not relevant in our cohort. This is in keeping with the fact that the literature to date has found significant genetic heterogeneity for susceptibility to KD amongst different racial groups. In fact, only SNPs in 3 genes (*ITPKC*, inositol 1,4,5 triphosphate 3-kinase C; *FcyR2A*, FcGamma receptor 2A; *CASP3*, caspase-3) have been validated in independent cohorts across multiple different racial populations [5]. SNPs in *ITPKC* and *CASP3* were significantly increased in our cohort but we did not find any significant difference when assessing the *FcyR2A* rs1801274 SNP.

### VEGFA

Multiple SNPs in *VEGFA* have been associated with KD and there appears to be variation in their relevance according to ethnicity [38, 39]. The *VEGFA* rs699947 (C/A) polymorphism occurs in the 5’ untranslated region of *VEGFA* promoter. This region facilitates the binding of activators and transcription factors and therefore rs699947 has a functional effect on vascular endothelial growth factor (VEGF) A expression. It has been shown that serum levels of VEGF-A are significantly increased in carriers of a rs699947 C allele [40].

In the current study we found that the frequency of the C/A alleles was 0.47/0.53 in the total population. This is comparable with European populations which displays a 0.45/0.55 frequency of rs699947 C/A. However, in analysis of our clinical outcomes, the frequency of the A allele decreases from 0.53 in the total population to 0.36 in patients with coronary artery aneurysms and 0.23 in patients with IVIG resistance. This demonstrates that presence of the C allele of rs699947 harbours increased risk, with carriers found to have an almost 3-times higher risk of coronary artery aneurysm formation and a 4-times higher risk of IVIG resistance, suggesting that the polymorphism is protective. This protective effect may be related to reduced VEGF production. Interestingly, in another European KD cohort, those with the CC genotype had higher mean VEGF plasma levels than those with AC or AA genotypes [41]. This SNP was not associated with susceptibility to KD or to CAL development in a cohort of Taiwanese children [42], suggesting that it is perhaps more relevant in Caucasian populations, although this requires further study. Although this SNP has been studied extensively in the vasculitis literature [43], the authors did not find any other studies examining it in Kawasaki disease.

In KD, immune system activation and cytokine production trigger an inflammatory cascade. The resulting inflammatory mediators directly damage vascular endothelial cells which may induce VEGF release. VEGF is a signal protein that promotes angiogenesis [44] and VEGFA is one of its isoforms. It is proinflammatory, via inducing the expression of endothelial adhesion molecules thus promoting monocyte and macrophage chemotaxis [45–48], and induces vascular permeability mediated by increased calcium influx [44].

VEGF has been implicated in both the acute phase of coronary vessel damage in KD and in the late chronic vascular remodeling which leads to progressive coronary stenosis. In the acute phase, VEGF levels are increased in patients with KD compared to afebrile controls or patients with infectious diseases [49–55]. Serum VEGF level at diagnosis is also an independent major risk factor for coronary artery lesions [51]. In a murine model of coronary arteritis, there was an association between local VEGFA and its signaling pathways and the development of CALs [56]. Regarding late remodeling, an immunohistochemical study found extensive expression of VEGF in proliferating smooth muscle cells of the intima and microvasculature in coronary arteries of patients with a history of KD. In the control cohort, VEGF was expressed in the medial smooth muscle cells but not in the intimal layer [57].

IVIG, even when given with dexamethasone, does not seem to decrease VEGF levels [52, 58]. Studies have shown that other treatments such as infliximab dramatically decrease the levels of other pro-inflammatory cytokines but not VEGF suggesting that these therapies may not be effective at stopping local coronary damage [59]. Determining the exact role of VEGF in the pathophysiology of KD is crucial to understanding whether VEGF antagonists may play a role in treatment.

### CD40

CD40 is a transmembrane glycoprotein and a member of the tumor necrosis factor superfamily. It is expressed on a wide range of cells including antigen presenting cells, B cells and monocytes. CD40 plays an important role in amplification of the immune response and in B cell activation [26]. GWAS have repeatedly shown it to be one of the most significant susceptibility genes for KD [27, 28]. *CD40* is known as a susceptibility gene for multiple adult autoimmune diseases (including rheumatoid arthritis and systemic lupus erythematous). The SNPs for these diseases belong to the same SNP group of high linkage disequilibrium as for KD and are thought to enhance B cell activity [27]. We tested 4 SNPs of the *CD40* gene which had previously been associated with KD in predominantly Asian populations [60]: rs1535045, rs1569723, rs4810485, rs4813003. The rs1569723 was significant in our patient cohort. To the best of our knowledge, this is the first time this SNP has been associated with KD susceptibility and clinical outcomes in a Caucasian population.

We found the *CD40* rs1569723 (C/A) was significantly associated with coronary lesions and IVIG resistance, where presence of the C allele led to a four-fold increased risk for coronary artery aneurysm formation and an almost eight-fold increased risk for IVIG resistance. The functional impact of rs1569723 has yet to be determined but it is known to be in complete linkage disequilibrium with another *CD40* polymorphism rs1883832 [61] which has been associated with increased levels of scavenger CD40 ligand [62].

### CRP

We chose CRP as a biomarker to study as we hypothesized that its association with clinical outcomes could be mediated via genetic variation. In keeping with previous studies [63–66], we found a trend towards higher CRP levels in patients with coronary artery lesions and IVIG resistance. This did not meet statistical significance which may have been due to the small sample size. However, we did not find a correlation between CRP levels and the polymorphisms (VEGFA rs699947 C/A, CD40 rs1569723 A/C) which were associated with risk of coronary artery lesions and IVIG resistance in our cohort.

### Implications

The genetics of KD have been studied since the late 1970s and arguably, although hypothesis generating, the clinical application of genetic studies has been limited to date. However, there are important implications of our work in the current era due to the advent of artificial intelligence and due to the occurrence of PIMS. We now know that early treatment intensification can improve outcomes in those at high risk of IVIG resistance. By harnessing powerful machine learning methods, there is an opportunity to incorporate genetic data (for example in the form of a high-risk SNP profile) in order to predict patients who will show IVIG resistance. Machine learning algorithms using clinical data have already demonstrated superior ability to predict IVIG resistant patients compared to previous clinical scoring models [67].

The occurrence of PIMS during the COVID pandemic elicited huge interest from the KD scientific community. There is still heated debate regarding whether PIMS and KD represent separate disease entities or are part of the same spectrum. Although they share many overlapping clinical features, and appear to share a common host immune response [68], one key difference is the age of children affected, with PIMS typically affecting older children than KD. It remains to be determined whether they share a common genetic predisposition.

## Limitations

We acknowledge the limitations of a small cohort size and potential selection bias. As ours is the only paediatric cardiology unit in Ireland, we likely identified the majority of patients who fulfilled the inclusion criteria. Some patients may have had echocardiograms in private hospitals or outreach clinics and may have been treated in peripheral units and therefore not been identified. We acknowledge that our patient cohort is likely to be biased towards the more severe end of the Kawasaki disease spectrum as severely affected patients are the most likely to be transferred to the national centre. This is reflected by the relatively high prevalence of coronary artery aneurysms and IVIG resistance in our cohort. While this may limit generalisability, we believe that reporting novel and significant findings—such as the association of the VEGF SNP with KD outcomes—is valuable and important. A recent review by Jane Burns [5] discusses genetic factors in KD and notes that some variants may be important only within families or within subgroups of patients. This supports the fact that it is worthwhile reporting on genetic variations even in small patient numbers, particularly when the findings are significant, as they are hypothesis generating, may lead to future research in the area and may also stimulate multi-centre collaborations in the future. Due to the study being carried out during the COVID pandemic and with an aim of reducing unnecessary patient contact, we asked study participants to carry out their buccal swabs at home and post the samples to our laboratory. Unfortunately, many of the samples subsequently did not amplify for every SNP during genotyping and this resulted in missing data. Of note the reference ALFA database also includes variation in sample sizes across different SNPs. Future studies should aim to collect blood or saliva samples to enhance DNA yield and quality, which will likely increase the SNP sequencing call rate.

It should be noted that three patients did not receive IVIG due to late presentation. We excluded these patients from the analysis for IVIG resistance but included them when analysing association with coronary outcomes. Although treatment with IVIG is a major modifying factor for development of coronary complications, it is not the sole determinant, and genetics are postulated to play an important role [27]. Therefore, we believed it appropriate to include these patients in the analysis.

## Conclusion

Despite decades of research, the underlying pathophysiological mechanisms of Kawasaki disease remain incompletely understood. A complex genetic pattern likely impacts susceptibility to both KD, coronary artery aneurysm development and IVIG resistance and these patterns are heterogenous amongst different ethnic groups. We performed the first evaluation of the genetic profile of patients with Kawasaki disease in Ireland by assessing for the presence of SNPs associated with KD susceptibility and outcomes in other populations. We found that the *VEGF* rs699947 C/A polymorphism is protective and associated with a decreased risk of coronary artery lesions and IVIG resistance while the *CD40* rs1569723 A/C polymorphism harbours increased risk. In the era of machine learning, future research should evaluate whether an individual’s SNP genotype profile and clinical data could identify high-risk patients who would benefit from early treatment intensification with adjunctive or second-line agents.

## Supplementary Information

Below is the link to the electronic supplementary material.

Supplementary file1 (DOCX 19 KB)

## Data Availability

All data supporting the findings of this study are available within the paper and its Supplementary Information.

## Abbreviations

CAA: Coronary artery aneurysm
CRP: C-reactive protein
IVIG: Intravenous immunoglobulin
KD: Kawasaki disease
SNP: Single nucleotide polymorphism
