# Supplementary material for: Single Nucleotide Polymorphisms and Their Association with Coronary Artery Aneurysms and IVIG Resistance in Kawasaki Disease in Ireland
Source: Pediatr Cardiol. 2025 Aug 8;47(5):2018–28. doi: 10.1007/s00246-025-03989-0 (PMC13144236; doi:10.1007/s00246-025-03989-0)
Supplement: Supplementary file 1 — Supplementary file1 (DOCX 19 KB) [file 246_2025_3989_MOESM1_ESM.docx]

Supplementary Table 1 Comparison of prevalence of SNPs selected for susceptibility to KD in our cohort compared to a European Reference Cohort

|  |  |  | **Kawasaki Disease Cohort** | | | | | **European Population** | | | | |  |  |
| --- | --- | --- | --- | --- | --- | --- | --- | --- | --- | --- | --- | --- | --- | --- |
|  |  |  | Sample size | Ref | | Alt. | | Sample size | Ref | | Alt | | P value | P star |
| SMAD5 | rs10056474 | C > G | 32 | C | 0.59375 | G | 0.40625 | 10410 | C | 0.83977 | G | 0.16023 | 0.0008 | *** |
| CASP3 | rs113420705 | A > G | 14 | A | 0.428571 | G | 0.571429 | 30854 | T | 0.71812 | C | 0.28188 | 0.0314 | * |
| CD40 | rs1535045 | C > T | 20 | C | 0.7000 | T | 0.30000 | 11062 | C | 0.84361 | T | 0.15639 | 0.1129 | ns |
| CD40 | rs1569723 | C > A | 34 | C | 0.441176 | A | 0.558824 | 291418 | C | 0.25962 | A | 0.74038 | 0.0289 | * |
| FCGR2A | rs1801274 | A > G | 26 | A | 0.461538 | G | 0.538462 | 305336 | A | 0.508302 | G | 0.49170 | 0.6973 | ns |
| CCL17 | rs223895 | T > C | 48 | T | 0.25000 | C | 0.75000 | 172832 | T | 0.333844 | C | 0.666156 | 0.2835 | ns |
| ITPKC | rs2290692 | G > C | 40 | G | 0.30000 | C | 0.70000 | 9602 | G | 0.5941 | C | 0.39610 | 0.0001 | *** |
| BLK | rs2736340 | C > T | 68 | C | 0.720588 | T | 0.279412 | 298204 | C | 0.751174 | T | 0.248826 | 0.575 | ns |
| ITPKC | rs28493229 | G > C | 70 | G | 0.871429 | C | 0.128571 | 75010 | G | 0.87104 | C | 0.12896 | >0.9999 | ns |
| TGFBR2 | rs3773649 | G > A | 26 | G | 0.769231 | A | 0.230769 | 282418 | G | 0.714406 | A | 0.285594 | 0.6664 | ns |
| CD40 | rs4810485 | T > G | 26 | T | 0.307692 | G | 0.692308 | 184008 | T | 0.262836 | G | 0.737164 | 0.6559 | ns |
| CD40 | rs4813003 | C > T | 26 | C | 0.769231 | T | 0.230769 | 189932 | C | 0.858423 | T | 0.141577 | 0.2521 | ns |
| VEGFA | rs699947 | A > C | 66 | A | 0.530303 | C | 0.46970 | 19174 | A | 0.55226 | C | 0.44774 | 0.8044 | ns |
| SMAD3 | rs7163381 | A > G | 22 | A | 0.318182 | G | 0.681818 | 144076 | A | 0.259044 | G | 0.740956 | 0.4768 | ns |
| ITPKC | rs7251246 | C > T | 52 | C | 0.403846 | T | 0.596154 | 18126 | C | 0.50883 | T | 0.49117 | 0.1643 | ns |
| GRIN3A | rs7849782 | C > G | 18 | C | 0.388889 | G | 0.611111 | 6778 | C | 0.62200 | G | 0.37800 | 0.0515 | ns |
